# The evaluation of frequency and risk factors of house dust mites allergy in obese children

**DOI:** 10.1186/1939-4551-8-S1-A79

**Published:** 2015-04-08

**Authors:** Eliza Wasilewska, Sylwia Malgorzewicz

**Affiliations:** 1 Medical University of Gdansk, Poland

## Background

During last years, more frequently increase of number of school-age children with obesity was observed in the European and American societies. The increased body mass has numerous health consequences, including impairment function of the respiratory and immunology system.

## Aim

The aim of the study was to evaluate the frequency and risk factors for dust mites allergy in children with obesity.

## Methods

103 school age children (7-16 y.o. M 50, F 53) with obesity (centile of BMI>97) were included to the study. All children were under the care of the out-patient endocrinology department. Clinical data, detail interview about the risk factors of obesity and allergies, were evaluated. Prick skin tests (*Allergopharma*) with common alergens and spirometry (*Jaeger*) were performed in all children. The statistical analysis was done using program Statistica vs 10.0.

## Results

23 (22%) children had HDMA. 16 (70%) children with HDMA presented symptoms of asthma and allergic rhinitis, 6 (26%) only rhinitis. Patiens without HDMA (nHDMA): 2 (3%) had rhinitis chronica, 6 (8%) asthma, 5 (6%) had both asthma and rhinitis chronica. Children with HDMA had more frequently rhinitis chronica than nHDMA children (p<0,05) and asthma with rhinitis chronica (p<0.05). The age, brestfeeding, parents obesity, coexisting of thyroid and hyperinsulinemic disturbances, type of obesity, exposition to house animals, did not significantly correlating with HDMA. The parents allergies increased the risk of HDMA in obesity children (p<0.05).

## Conclusions

23% school aged obese children had HDMA. The most frequency of clinical manifestation of HDMA in obese children was asthma with rhinitis chronica (70%). The parents allergy was a risk factor for HDMA. A screening for respiratory abnormalities and HDMA in obese children, seems to be of significant importance.

